# Supplementary material for: Choline Is an Intracellular Messenger Linking Extracellular Stimuli to IP3-Evoked Ca2+ Signals through Sigma-1 Receptors
Source: Cell Rep. 2019 Jan 8;26(2):330–337.e4. doi: 10.1016/j.celrep.2018.12.051 (PMC6326163; doi:10.1016/j.celrep.2018.12.051)
Supplement: Document S1. Figures S1 and S2 [file mmc1.pdf]

Cell Reports, Volume *26*

Supplemental Information

Choline Is an Intracellular Messenger Linking Extracellular Stimuli to IP_3-Evoked Ca^{2+} Signals through Sigma-1 Receptors

Eugen Brailoiu, Sumita Chakraborty, G. Cristina Brailoiu, Pingwei Zhao, Jeffrey L. Barr, Marc A. Ilies, Ellen M. Unterwald, Mary E. Abood, and Colin W. Taylor

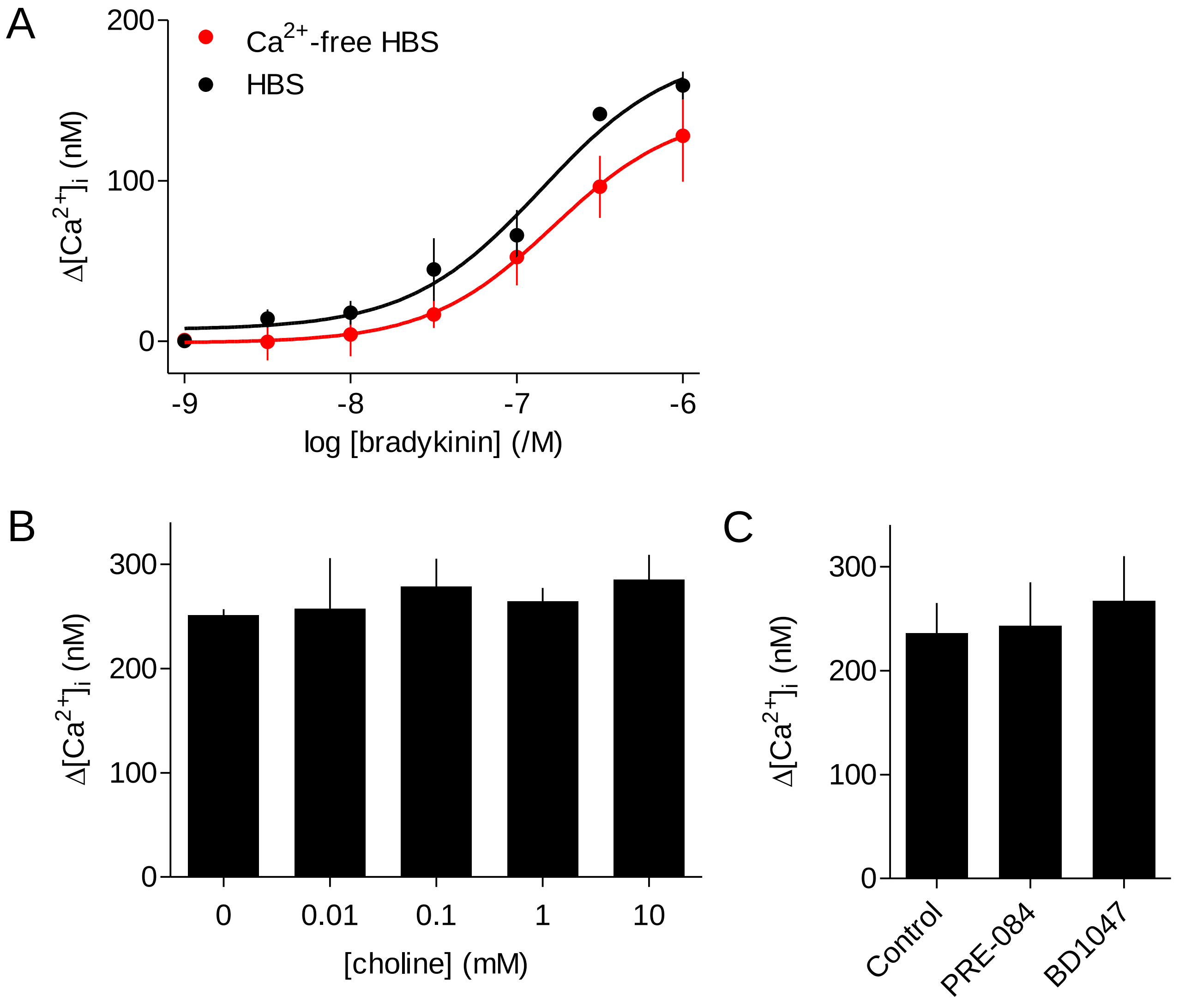

Figure S1. Bradykinin Stimulates Ca^{2+} Release From the Intracellular Stores of NG108-15 Cells.
Related to Figures 1 and 2

(A) NG108-15 cells were stimulated with the indicated concentrations of bradykinin in HBS or Ca^{2+}-free HBS. Results (means ± SEM, n = 3, with duplicate determination) show $\Delta[Ca^{2+}]_i$.
(B,C) Intact cells were treated with the indicated concentrations of choline (1 h, 20°C) (B) or incubated (3 h, 37°C) with PRE-084 (25 μM) or BD1047 (25 μM) (C) before addition of ionomycin (5 μM) in Ca^{2+}-free HBS to assess the Ca^{2+} content of the intracellular stores. Results (mean ± SEM, n = 3) show $\Delta[Ca^{2+}]_i$ evoked by ionomycin.

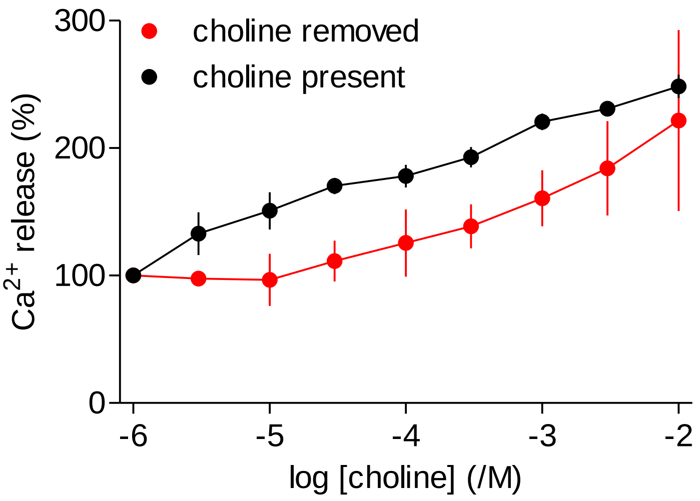

Figure S2. Intracellular Choline Potentiates Bradykinin-Evoked Ca^{2+} Signals. Related to Figure 4.

NG108-15 cells were incubated (1 h) with the indicated concentrations of choline in HBS before recording the peak Ca^{2+} signals evoked by bradykinin (1 µM), which was added in either the continued presence of choline or immediately after its removal. Responses (mean ± SEM, *n* = 3 plates with duplicate determinations; several error bars are smaller than the symbols) are expressed as a percentage of the matched response to bradykinin without choline (100%).
