# Supplementary material for: Choline Is an Intracellular Messenger Linking Extracellular Stimuli to IP3-Evoked Ca2+ Signals through Sigma-1 Receptors
Source: Cell Rep. 2019 Jan 8;26(2):330–337.e4. doi: 10.1016/j.celrep.2018.12.051 (PMC6326163; doi:10.1016/j.celrep.2018.12.051)
Supplement: Document S2. Article plus Supplemental Information [file mmc2.pdf]

Cell Reports

Report

Choline Is an Intracellular Messenger Linking Extracellular Stimuli to IP_3-Evoked Ca^{2+} Signals through Sigma-1 Receptors

Graphical Abstract

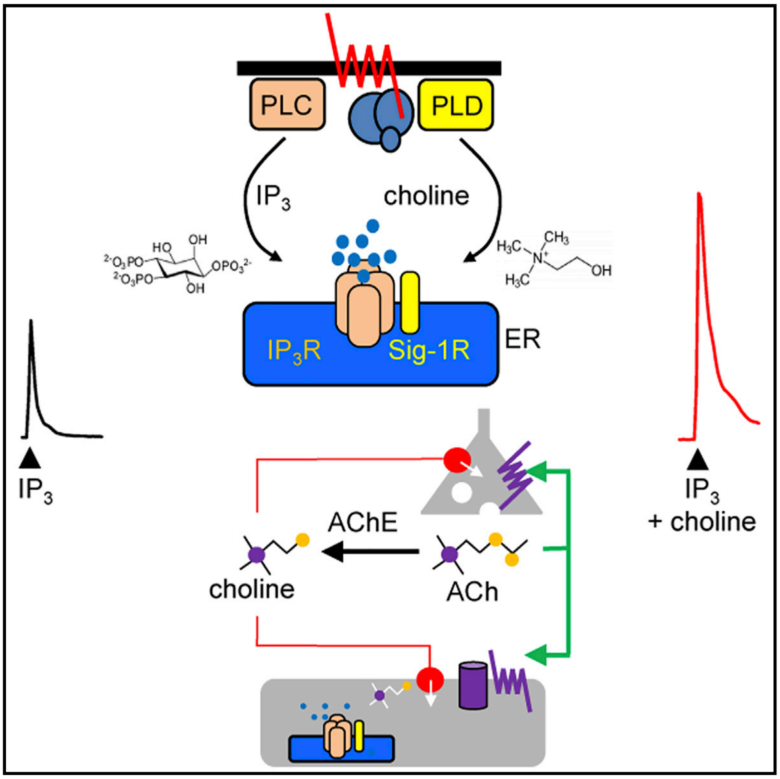

Highlights

- Choline, but not its metabolites, binds to Sigma-1 receptors
- Choline potentiates IP_3-evoked Ca^{2+} release by activating Sigma-1 receptors
- Bradykinin stimulates Ca^{2+} release by stimulating formation of IP_3 and choline
- Choline uptake by a specific transporter potentiates IP_3-evoked Ca^{2+} release

Authors

Eugen Brailoiu, Sumita Chakraborty, G. Cristina Brailoiu, ..., Ellen M. Unterwald, Mary E. Abood, Colin W. Taylor

Correspondence

ebrailou@temple.edu (E.B.), cwt1000@cam.ac.uk (C.W.T.)

In Brief

Sigma-1 receptors respond to diverse stimuli and regulate many signaling proteins. Brailoiu et al. show that choline is an endogenous agonist of Sigma-1 receptors. Choline links receptors and cholinergic synaptic activity, through Sigma-1 receptors, to enhanced Ca^{2+} release through IP_3 receptors.

Brailoiu et al., 2019, Cell Reports *26*, 330–337
January 8, 2019 © 2018 The Authors.
https://doi.org/10.1016/j.celrep.2018.12.051

OPEN ACCESS
CellPress

Cell Reports
Report

Choline Is an Intracellular Messenger Linking Extracellular Stimuli to IP_3-Evoked Ca^{2+} Signals through Sigma-1 Receptors

Eugen Brailoiu,[1,6,*] Sumita Chakraborty,[2,5,6] G. Cristina Brailoiu,[3] Pingwei Zhao,[1] Jeffrey L. Barr,[1] Marc A. Ilies,[4] Ellen M. Unterwald,[1] Mary E. Abood,[1] and Colin W. Taylor[2,7,*]
[1]Center for Substance Abuse Research, Lewis Katz School of Medicine, Temple University, Philadelphia, PA 19140, USA
[2]Department of Pharmacology, Tennis Court Road, Cambridge CB2 1PD, UK
[3]Department of Pharmaceutical Sciences, Jefferson College of Pharmacy, Thomas Jefferson University, Philadelphia, PA 19107, USA
[4]Department of Pharmaceutical Sciences, Temple University School of Pharmacy, Philadelphia, PA 19140, USA
[5]Present address: Centre for Brain Development and Repair, Institute for Stem Cell Biology and Regenerative Medicine, Bengaluru, KA 560065, India
[6]These authors contributed equally
[7]Lead Contact
*Correspondence: ebrailou@temple.edu (E.B.), cwt1000@cam.ac.uk (C.W.T.)
https://doi.org/10.1016/j.celrep.2018.12.051

SUMMARY

Sigma-1 receptors (Sig-1Rs) are integral ER membrane proteins. They bind diverse ligands, including psychoactive drugs, and regulate many signaling proteins, including the inositol 1,4,5-trisphosphate receptors (IP_3Rs) that release Ca^{2+} from the ER. The endogenous ligands of Sig-1Rs are unknown. Phospholipase D (PLD) cleaves phosphatidylcholine to choline and phosphatidic acid (PA), with PA assumed to mediate all downstream signaling. We show that choline is also an intracellular messenger. Choline binds to Sig-1Rs, it mimics other Sig-1R agonists by potentiating Ca^{2+} signals evoked by IP_3Rs, and it is deactivated by metabolism. Receptors, by stimulating PLC and PLD, deliver two signals to IP_3Rs: IP_3 activates IP_3Rs, and choline potentiates their activity through Sig-1Rs. Choline is also produced at synapses by degradation of acetylcholine. Choline uptake by transporters activates Sig-1Rs and potentiates Ca^{2+} signals. We conclude that choline is an endogenous agonist of Sig-1Rs linking extracellular stimuli, and perhaps synaptic activity, to Ca^{2+} signals.

INTRODUCTION

The Sigma-1 receptor (Sig-1R) is a small integral membrane protein expressed mainly in the endoplasmic reticulum (ER) and concentrated at the dynamic contacts between mitochondria and ER, the mitochondria-associated ER membrane domains (MAMs) (Schmidt et al., 2016; Smith and Su, 2017; Su et al., 2016). Sig-1R was thought to have two transmembrane domains (TMDs), with its N and C termini in the ER lumen (Aydar et al., 2002; Hayashi and Su, 2007). This topology was consistent with evidence that BiP, an ER luminal chaperone protein, binds to the C-terminal domain of Sig-1R (Hayashi and Su, 2007). However, a crystal structure of Sig-1R challenges these observations because it identified only a single TMD within each subunit of a trimeric complex, and it placed the C-terminal region on the cytosolic side of the ER membrane (Alon et al., 2017; Schmidt et al., 2016).

Sig-1Rs are abundant in brain, but they are also expressed in other tissues (Smith and Su, 2017). They are implicated in many pathologies, including depression, anxiety, amyotrophic lateral sclerosis and other neurodegenerative diseases, drug addiction, neuropathic pain, and cancers (Gueguinou et al., 2017; Su et al., 2016; Watanabe et al., 2016). Sig-1Rs bind an unusually diverse array of ligands, most of which are amines. These include antidepressants (e.g., fluoxetine), antipsychotics (e.g., haloperidol), and drugs of abuse (e.g., cocaine and methamphetamine) (Maurice and Su, 2009; Walker et al., 1990). Sig-1Rs also interact with many different signaling proteins. Within the ER, these proteins include inositol 1,4,5-trisphosphate receptors (IP_3Rs) (Hayashi and Su, 2007) and STIM1, the Ca^{2+} sensor for store-operated Ca^{2+} entry (Srivats et al., 2016). At the plasma membrane (PM), Sig-1Rs regulate a variety of receptors and ion channels (Su et al., 2016).

Although many ligands of Sig-1Rs have opposing effects, their diversity and the many proteins that interact with Sig-1Rs confound attempts to classify ligands consistently as agonists or antagonists across all bioassays (Schmidt et al., 2016; Yano et al., 2018). A more fundamental distinction may be whether ligands stabilize oligomeric (antagonists) or monomeric forms (agonists) of Sig-1R (Gromek et al., 2014; Mishra et al., 2015; Ossa et al., 2017; Schmidt et al., 2016; Yano et al., 2018). Hence, agonists by releasing Sig-1Rs from large oligomeric complexes may free Sig-1Rs to interact with client proteins (Figure 1A). Several endogenous molecules, including steroids (Monnet and Maurice, 2006) (notably progesterone), various sphingolipids (Ramachandran et al., 2009), and *N*,*N*-dimethyltryptamine (DMT) (Fontanilla et al., 2009), bind to Sig-1Rs and regulate some of their activities. It is unclear whether any of these ligands mediate endogenous regulation of Sig-1Rs, and none has been shown to link extracellular stimuli to regulation of Sig-1Rs.

 © 2018 The Authors.
This is an open access article under the CC BY license (http://creativecommons.org/licenses/by/4.0/).

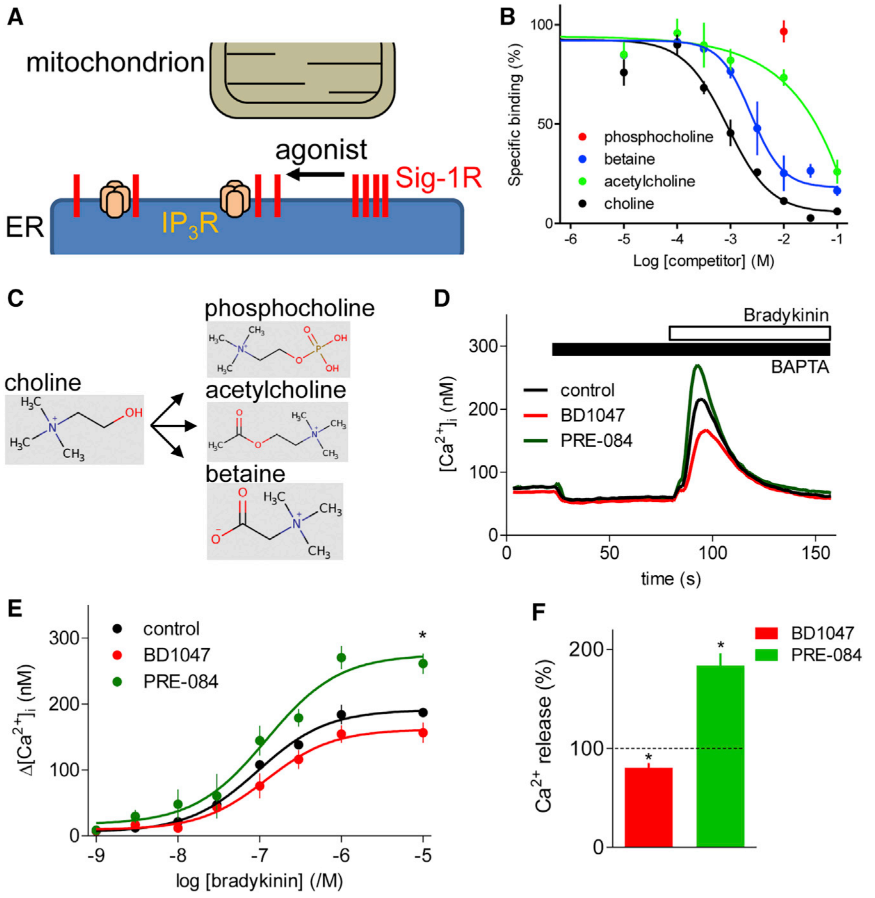

Figure 1. Choline Is an Agonist of Sig-1Rs

(A) Clusters of Sig-1Rs anchored at MAMs are thought to dissociate into monomers when they bind a Sig-1R agonist, freeing Sig-1Rs to interact with their targets, within and beyond MAMs. The targets include IP_3Rs.

(B) Specific binding of [^{3}H](+)-pentazocine (5 nM) in the presence of choline and related compounds using membranes from Neuro-2A cells stably expressing Sig-1R-GFP (mean ± SEM; n = 5, with 3 replicates for each). Specific binding of ^{3}H-pentazocine was 90% ± 3% of total binding (mean ± SEM; n = 3) for membranes from cells overexpressing Sig-1R, and 13% ± 5% for mock-transfected cells.

(C) Choline metabolism (structures from http://www.hmdb.ca).

(D) NG108-15 cells were incubated (2 hr, 37°C) with PRE-084 (25 μM) or BD1047 (25 μM), and then, in the continuous presence of the Sig-1R ligands, loaded with Fluo-8 by incubation with Fluo-8 AM in HEPES-buffered saline (HBS) (30 min, 20°C, with a further 30 min to allow de-esterification of Fluo-8). BAPTA (2.5 mM) was then added to chelate extracellular Ca^{2+} before addition of bradykinin (10 μM). Results show typical responses as means of 3 replicates.

(E) Summary results (mean ± SEM; n = 5, each with 3 replicates) show peak increases in $[Ca^{2+}]_i$ ($\Delta[Ca^{2+}]_i$) evoked by bradykinin. *$p < 0.05$ for maximal responses relative to control, one-way ANOVA with Dunnett's test.

(F) Pooled results (mean ± SEM; n = 20; as percentages of matched control response) for all bradykinin concentrations. The asterisk (*) denotes 95% confidence intervals that exclude 100%.

See also Figure S1A.

Many extracellular stimuli evoke increases in the intracellular free $[Ca^{2+}]$ ($[Ca^{2+}]_i$) through receptors that stimulate phospholipase C (PLC), leading to formation of IP_3 and release of Ca^{2+} from the ER through IP_3Rs. Sig-1Rs have been reported to both potentiate the Ca^{2+} signals evoked by these receptors by increasing the IP_3 sensitivity of IP_3Rs (Hayashi et al., 2000; Hong et al., 2004; Wu and Bowen, 2008) and to increase the efficiency of Ca^{2+} transfer from ER to mitochondria through IP_3Rs (Hayashi and Su, 2007; Shioda et al., 2012).

Here, we demonstrate that agonists of G-protein-coupled receptors (GPCRs) that stimulate PLC and an increase in $[Ca^{2+}]_i$, also stimulate phospholipase D (PLD). We show that choline produced by PLD is an endogenous agonist of Sig-1Rs, and that it thereby potentiates Ca^{2+} signals evoked by IP_3Rs. Each of the immediate products of choline metabolism, phosphocholine, acetylcholine, and betaine, is inactive. Hence, GPCRs signal to IP_3Rs through two parallel pathways that converge to provide coincident stimulation of IP_3Rs. In addition, choline uptake by specific transporters allows extracellular choline to stimulate Sig-1Rs and potentiate IP_3-evoked Ca^{2+} signals. We conclude that choline is an endogenous agonist of Sig-1Rs that links both cell signaling pathways (through PLD) and the activity of cholinergic synapses (through choline uptake) to regulation of IP_3-evoked Ca^{2+} signals.

RESULTS

Choline Binds to Sig-1Rs and Potentiates IP_3-Evoked Ca^{2+} Signals

Most high-affinity ligands of Sig-1Rs comprise a tertiary amine flanked by a short acyl chain and hydrophobic moieties (Glennon, 2005; Ossa et al., 2017). Endogenous agonists are unlikely to have such high affinity because they must rapidly associate with and dissociate from Sig-1Rs if they are to acutely regulate them. We considered whether choline, a quaternary amine with an acyl chain but no hydrophobic moieties, might be an endogenous agonist of Sig-1Rs.

(+)-Pentazocine is a high-affinity, selective ligand of Sig-1Rs (equilibrium dissociation constant, K_d = 5.5 nM) (de Costa et al., 1989). Specific binding of [^{3}H](+)-pentazocine to membranes prepared from Neuro-2A cells stably expressing Sig-1R was completely displaced by choline (K_i = 525 μM; pK_i = 3.28 ± 0.16; h = 1.07 ± 0.2; mean ± SEM; n = 5; where pK_i is the negative log of the K_d, and h is the Hill coefficient) (Figure 1B). Phosphocholine, the major product of choline metabolism in most cells (Figure 1C) (Corbin and Zeisel, 2012), did not displace specific [^{3}H](+)-pentazocine from Sig-1Rs, and the other immediate products of choline metabolism, betaine (K_i = 1.32 mM; pK_i = 2.88 ± 0.23; h = 1.40 ± 0.35; n = 5) and acetylcholine (K_i ~12 mM; n = 5), were less

OPEN
ACCESS
CellPress

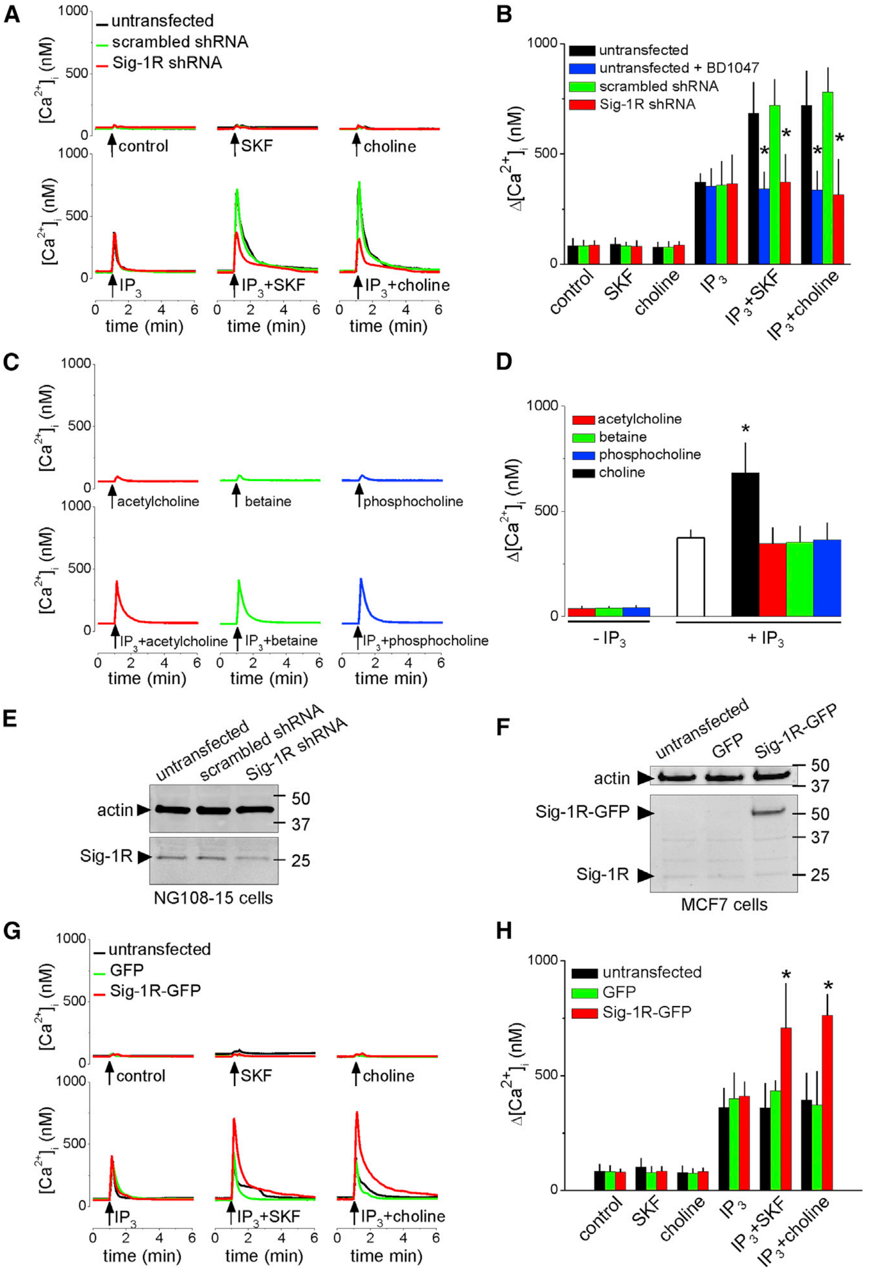

Figure 2. Choline Potentiates IP_3-Evoked Ca^{2+} Release by Stimulating Sig-1Rs

(A) Ca^{2+} signals recorded from Fura-2-loaded NG108-15 cells after microinjection (~1% cell volume) of IP_3 (pipette concentration, 0.5 μM), (+)SKF-10047 (SKF, 100 μM), or choline (100 mM). Results (n = 6 cells) show untransfected cells or after transfection with scrambled shRNA or Sig-1R shRNA, each tagged with red fluorescent protein (RFP).

(B) Summary (mean ± SD; n = 6) shows peak $[Ca^{2+}]_i$. *p < 0.05, ANOVA with Bonferroni test, relative to matched stimuli in untransfected cells. The effects of pre-incubating cells with BD1047 (25 μM, 15 min) are also shown.

(C) Similar analysis of the effects of microinjected IP_3 (pipette concentration, 0.5 μM) or acetylcholine, betaine, or phosphocholine (pipette concentration, 100 mM for each), alone or in combination.

(D) Summary (mean ± SD; n = 6) shows peak $[Ca^{2+}]_i$. *p < 0.05, ANOVA with Bonferroni test, relative to IP_3 alone.

(E) Western blot (WB) of Sig-1R after transfection of NG108-15 cells with scrambled or Sig-1R shRNA, each tagged with RFP. Tagged shRNAs were used to allow identification of transfected cells in microinjection experiments. Hence, WB from cell populations probably over-estimates Sig-1R expression in functional analyses of microinjected cells treated with Sig-1R shRNA. Sig-1R expression was reduced to 50% ± 12% of control levels by the shRNA treatment (mean ± SD; n = 3).

(F) WB showing detectable expression of Sig-1R in MCF7 cells only after transfection with Sig-1R-GFP. Typical of 4 blots. M_r markers (kDa) are shown.

(G) Ca^{2+} signals recorded from Fura-2-loaded MCF7 cells after microinjection as described for (C). Results (n = 6 cells) are from control cells or after transfection with GFP or Sig-1R-GFP.

(H) Summary (mean ± SD; n = 6) results show $[\Delta Ca^{2+}]_i$. *p < 0.05 for maximal responses relative to matched untransfected cells, one-way ANOVA with Dunnett's test.

See also Figures S1B and S1C.

effective than choline. This is consistent with choline binding with greater affinity than its metabolites to the same site as known agonists and antagonists of Sig-1Rs.

Subsequent experiments explore the interactions of choline with Sig-1Rs in NG108-15 cells. These neuroblastoma-glioma hybrid cells retain many properties of neurons, including responsiveness to neurotransmitters, and the ability to synthesize and release acetylcholine (Hamprecht et al., 1985); they express endogenous Sig-1Rs, and their bradykinin receptors stimulate PLC and Ca^{2+} release from the ER through IP_3Rs (Figure S1A). The bradykinin-evoked Ca^{2+} signals were enhanced by pre-incubation with a Sig-1R agonist (PRE-084) and attenuated by an antagonist (BD1047) (Figures 1D–1F). Microinjection of NG108-15 cells with IP_3 evoked a transient increase in $[Ca^{2+}]_i$, whereas microinjection of choline or the Sig-1R agonist, (+)SKF-10047, had no effect. However, co-injection of choline or (+)SKF-10047 with IP_3 potentiated the IP_3-evoked Ca^{2+} signals (Figures 2A and 2B). When applied to intact cells, neither choline nor other Sig-1R ligands significantly affected the Ca^{2+} content of the intracellular stores (Figures S1B and S1C). The potentiation of IP_3-evoked Ca^{2+} release by choline was blocked by pre-incubation with the Sig-1R antagonist, BD1047 (Figure 2B). Neither betaine, phosphocholine, nor acetylcholine mimicked the effects of microinjected choline (Figures 2C and 2D).

Treatment of NG108-15 cells with appropriate short hairpin RNA (shRNA) reduced expression of Sig-1R (Figure 2E) and

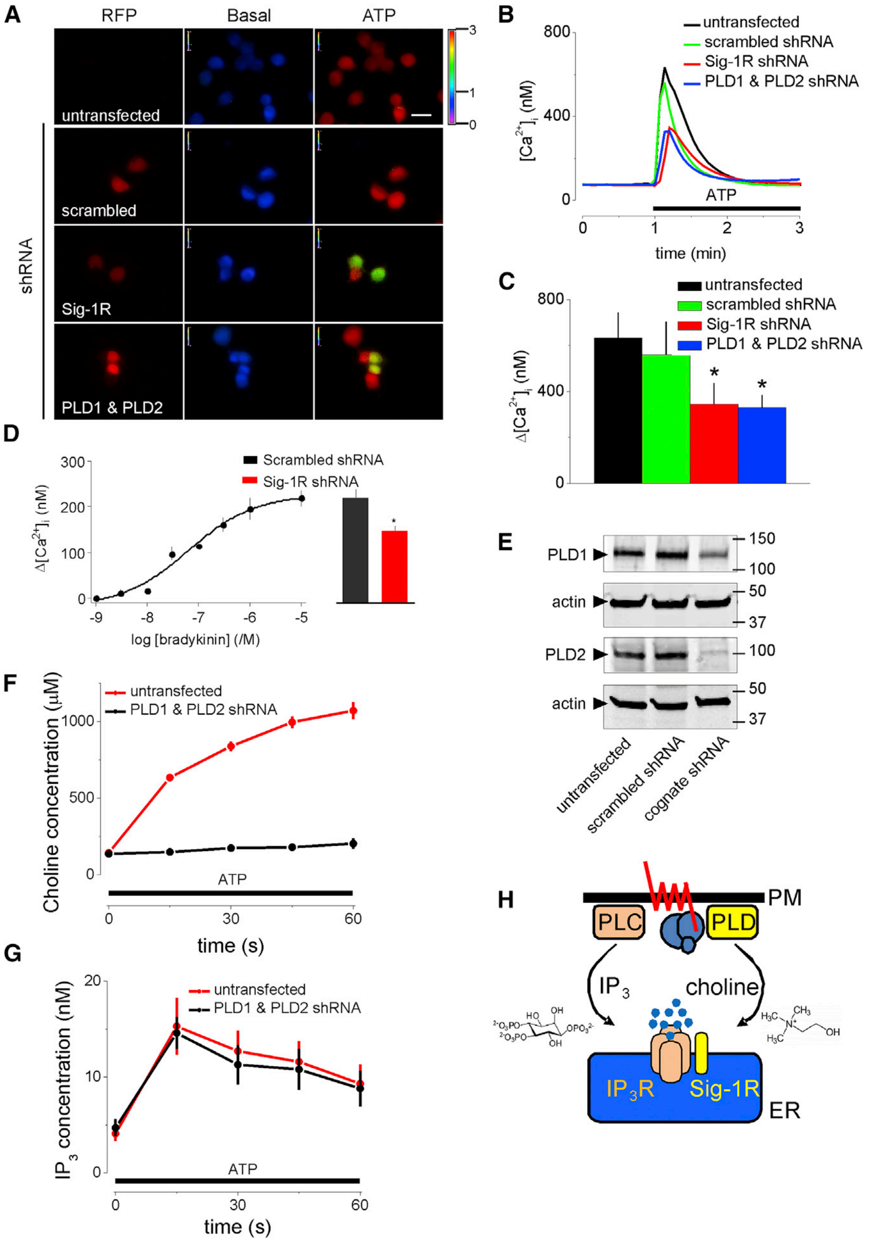

Figure 3. Sig-1R and PLD Contribute to Ca^{2+} Signals Evoked by Agonists of GPCRs

(A) Typical pseudocolor images show peak Ca^{2+} signals (F_{340}/F_{380}) evoked by ATP (50 μM) in Fura-2-loaded NG108-15 cells transfected with control shRNA or shRNA to PLD1 and PLD2, or Sig-1R, each tagged with RFP. Calibration code (F_{340}/F_{380}) and scale bar (20 μm) apply to all panels.

(B) Time course of response to ATP (bar; n = 6).

(C) Summary (mean ± SD; n = 6) shows $\Delta[Ca^{2+}]_i$ evoked by ATP. *$p < 0.05$, ANOVA with Bonferroni test, relative to untransfected cells.

(D) $\Delta[Ca^{2+}]_i$ evoked by bradykinin in populations of NG108-15 cells. Histogram (which shares the y axis) compares responses to bradykinin (10 μM) after treatment with scrambled or Sig-1R shRNA. Results are means ± SEM; n = 3 with duplicate determinations. *$p < 0.05$, Student's t test.

(E) WB shows effects of indicated shRNA, each tagged with RFP, on expression of PLD1 and PLD2 in NG108-15 cells. M_r markers (kDa) are shown. Results, typical of 3 WBs, underestimate knockdowns in the cells used for Ca^{2+} measurements, which used only cells shown to be transfected by expression of RFP (see A).

(F and G) Intracellular concentrations of choline (F) and IP_3 (G) during stimulation of NG108-15 cells with ATP (50 μM, bar) show the effects of shRNA for PLD1 and PLD2. Results show means ± SD; n = 6.

(H) GPCRs that activate PLC and phospholipase D (PLD) initiate two parallel signaling pathways that converge at IP_3Rs. IP_3 from PLC directly activates IP_3R. Choline from PLD activates Sig-1R, which potentiates IP_3-evoked Ca^{2+} release.

abolished the potentiating effects of choline and (+)SKF-10047, without affecting responses to IP_3 alone (Figures 2A and 2B). In MCF7 breast cancer cells, Sig-1R expression was scarcely detectable (Figure 2F) (Wu and Bowen, 2008). In these cells, neither microinjected choline nor (+)SKF-10047 potentiated IP_3-evoked Ca^{2+} signals, but the signals were potentiated after expression of Sig-1R-GFP (Figures 2F–2H). These results establish that choline, by activating Sig-1Rs, potentiates IP_3-evoked Ca^{2+} release.

Sig-1Rs Contribute to Ca^{2+} Signals Evoked by GPCRs

Extracellular ATP stimulates PLC through $P2Y_6$ receptors in NG108-15 cells (Sak et al., 2001). Loss of Sig-1Rs in NG108-15 cells (by shRNA) reduced the amplitude of the Ca^{2+} signals evoked by maximally effective concentrations of ATP (Figures 3A–3C) or bradykinin (Figure 3D). We next considered whether the contribution of Sig-1Rs to the Ca^{2+} signals evoked by GPCRs might be mediated by choline. Both mammalian isoforms of PLD (PLD1 and PLD2) are almost ubiquitously expressed enzymes that hydrolyse phosphatidylcholine (PC) to phosphatidic acid (PA) and choline. PLDs are regulated by many signals, including those that stimulate PLC and protein kinase C (PKC) (Selvy et al., 2011).

The basal choline concentration in NG108-15 cells (144 ± 7 μM) was similar to values reported for other cells (100–400 μM) (Pelech and Vance, 1984). Stimulation of NG108-15 cells with extracellular ATP increased the intracellular concentrations of both choline and IP_3. Knockdown of PLD1 and PLD2 expression using shRNA (Figure 3E) prevented the increase in choline concentration without affecting IP_3 production (Figures 3F and 3G). Furthermore, the ATP-evoked Ca^{2+} signals were similarly and substantially attenuated by loss of Sig-1R or loss of PLDs (Figures 3A–3C). The results so far demonstrate that GPCRs, by stimulating both

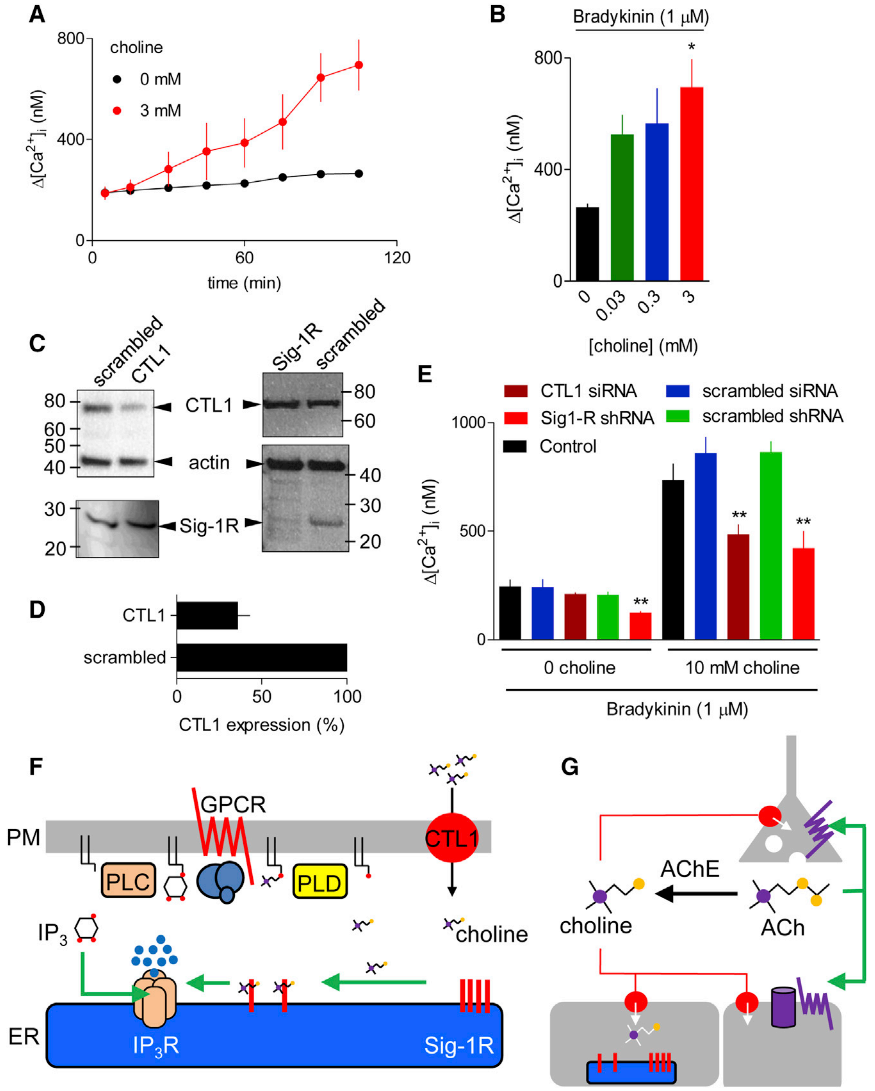

Figure 4. CTL1-Mediated Choline Uptake Potentiates IP_3-Evoked Ca^{2+} Signals

(A) NG108-15 cells were incubated in HBS alone or with 3 mM choline for the indicated times before adding bradykinin (1 μM) and immediately recording the increase in $[Ca^{2+}]_i$. Results (mean ± SEM; n = 3 with duplicate determinations) show $\Delta[Ca^{2+}]_i$ evoked by bradykinin.

(B) Summary results (mean ± SEM; n = 3) show bradykinin-evoked $\Delta[Ca^{2+}]_i$ after incubation with the indicated choline concentrations (105 min).

(C) WB showing effects of the indicated siRNA (for CTL1) or shRNA (for Sig-1R) and their scrambled counterparts on expression of CTL1 and Sig-1R in NG108-15 cells. M_r markers (kDa) are shown.

(D) Summary results (mean ± SD; n = 5) show CTL1 expression in cells treated with the indicated siRNA expressed as a percentage of the matched cells treated with scrambled siRNA.

(E) Summary results (mean ± SEM; n = 5 plates with 2 replicates) show the effects of 10 mM choline on bradykinin-evoked Ca^{2+} signals. *$p < 0.05$, **$p < 0.01$, one-way ANOVA with Dunnett's test, relative to control (B and E).

(F) Ca^{2+}-mobilizing GPCRs stimulate PLC and PLD, with consequent formation of IP_3 and choline. Although we have not resolved how GPCRs stimulate PLD in NG108-15 cells, signals evoked by both PLC and parallel pathways are known to stimulate PLD. IP_3 stimulates IP_3R, while choline binds to Sig-1Rs, causing them to potentiate IP_3R activity. Metabolism of IP_3 and choline terminates their signaling. Hence, GPCRs regulate IP_3Rs through two parallel, but converging, pathways. Import of extracellular choline by transporters, including the widely expressed CTL1, can also deliver choline to Sig-1Rs.

(G) Acetylcholine (ACh) released at cholinergic terminals can activate post- and pre-synaptic receptors, before its rapid hydrolysis to choline by acetylcholinesterase (AChE). Hence, synaptic activity is rapidly followed by a substantial local increase in choline concentration. Transporters (red circles) in the cholinergic terminal (CHT1) and neighboring cells (CTL1-5 and OCT) can import the choline, which will then stimulate Sig-1Rs, providing cells with a paracrine reporter of recent synaptic activity.

See also Figure S2.

PLC and PLD, generate parallel signals, IP_3 and choline, which converge to stimulate Ca^{2+} release through IP_3Rs (Figure 3H).

Choline Uptake Regulates Ca^{2+} Signals

Synthesis of acetylcholine within cholinergic nerve terminals requires choline uptake by a high-affinity, Na^+-dependent transporter (CHT1 [choline high-affinity transporter 1]) expressed mostly at cholinergic terminals (Haga, 2014; Sarter and Parikh, 2005). Additional Na^+-independent transporters mediate low-affinity choline uptake (OCTs [organic cation transporters]); and the widely expressed choline transporter-like proteins (CTL1-5, encoded by *SLC44A1-5*) mediate high-affinity uptake outside cholinergic terminals (Haga, 2014; Machová et al., 2009; Yamada et al., 2011). NG108-15 cells are capable of high-affinity choline uptake and they express CTL1, but not CHT1 (Machová et al., 2009), consistent with evidence that CTL1 is expressed in neurons and glia (Traiffort et al., 2013).

Incubation of NG108-15 cells with choline caused a time-dependent increase in the amplitude of the Ca^{2+} signals subsequently evoked by bradykinin (Figure 4A). The effect was minimally affected by removing the extracellular choline immediately before stimulation with bradykinin (Figure S2), suggesting that choline potentiates Ca^{2+} signals after its transport into cells. Potentiation of bradykinin-evoked Ca^{2+} signals by extracellular choline was substantially attenuated by loss of Sig-1R (shRNA) or CTL1 (small interfering RNA [siRNA]), but unaffected by scrambled shRNA or siRNA (Figures 4C–4E).

DISCUSSION

Sig-1Rs respond to many diverse drugs, including some that are commonly abused or used clinically, but it is unclear whether endogenous agonists regulate Sig-1Rs (Maurice and Su, 2009). Here, we provide evidence that choline (Figure 1C), best known as a precursor for synthesis of acetylcholine and PC, the most abundant membrane phospholipid in mammalian cells, is an endogenous agonist of Sig-1Rs. We show that choline meets the three essential criteria of an intracellular messenger, namely it is produced in response to extracellular stimuli, it exerts a specific intracellular action, and it is endogenously deactivated. We conclude that choline is an intracellular messenger linking GPCRs, through Sig-1Rs, to Ca^{2+} release from intracellular stores (Figure 4F).

Choline mimicked known Sig-1R agonists by competing with (+)-pentazocine for binding to Sig-1Rs (Figure 1B) and by potentiating the Ca^{2+} signals evoked by receptors that stimulate IP_3 formation (Figures 2A, 2B, and 2D). The immediate metabolites of choline were ineffective (Figures 2C and 2D). The effect of choline on Ca^{2+} signals was attenuated when Sig-1R expression was reduced (Figures 2A, 2B, and 2E); and in cells without Sig-1Rs, expression of Sig-1R endowed the cells with sensitivity to choline (Figures 2F–2H). The Ca^{2+} signals evoked by GPCRs that stimulate formation of IP_3 were attenuated when Sig-1R expression was reduced (Figures 3A–3D). ATP, which stimulates PLC through $P2Y_6$ receptors in NG108-15 cells (Sak et al., 2001), rapidly evoked formation of IP_3 and choline, but only the latter required PLDs (Figures 3F and 3G). Furthermore, the ATP-evoked Ca^{2+} signals were similarly attenuated by loss of PLDs or Sig-1Rs (Figure 3C). Bradykinin-evoked Ca^{2+} signals were likewise attenuated by loss of Sig-1Rs (Figure 3D).

Many GPCRs that stimulate PLC also activate PLD, and most agonists that activate PLD also stimulate PLC. However, the links between GPCRs and stimulation of mammalian PLD differ between cell types, and the stimulatory signals, which include PKC, Ca^{2+}, small GTPases (rho and ADP-ribosylation factor [Arf]), phosphatidylinositol 4,5-bisphosphate, and phosphatidylinositol 3,4,5-trisphosphate, can be generated by PLC or parallel pathways (Exton, 1999; Selvy et al., 2011). Hitherto, signaling downstream of PLD has been thought to arise entirely, directly or indirectly, from PA (Selvy et al., 2011). We suggest that the other product of PLD activity, namely choline, is also an important intracellular messenger that regulates Sig-1Rs and thereby IP_3-evoked Ca^{2+} release (Figures 3H and 4G). Our estimate of the intracellular choline concentration in NG108-15 cells after GPCR activation ($\sim$900 μM) (Figure 3F) is similar to that required for binding to Sig-1Rs (K_i = 525 μM) (Figure 1B). The low affinity of choline, relative to the many ligands used to establish structure-affinity relationship for Sig-1R (Glennon et al., 1992), is important because it will allow Sig-1R to respond rapidly to acute changes in intracellular choline concentration. We conclude that choline is an endogenous agonist of Sig-1Rs, a consequence of which includes potentiation of IP_3-evoked Ca^{2+} release (Figure 3H).

Choline is an essential nutrient that cells import through transporters from plasma, where the choline concentration is typically 5–10 μM, although it varies with diet (Sarter and Parikh, 2005). At cholinergic synapses, the choline concentration may be much higher ($\sim$1 mM) after synaptic activity, when acetylcholine is rapidly hydrolysed by acetylcholinesterase (Figure 4G). Our results show that extracellular choline, at concentrations encompassing likely synaptic concentrations, potentiates GPCR-evoked Ca^{2+} signals. The potentiation requires both Sig1R and the choline transporter, CTL1 (Figures 4A–4E and S2). These observations suggest an additional signaling role, whereby changes in extracellular choline concentration might regulate Sig-1Rs and thereby Ca^{2+} signaling. Such a mechanism might be particularly effective at cholinergic synapses of neuromuscular junctions or within the autonomic nervous system (Picciotto et al., 2012), where rapid transient increases in choline concentration follow synaptic activity. Choline might then determine the sensitivity of adjacent neurons or glia to PLC-coupled GPCRs (Figure 4G), consistent with many reported interactions between Sig-1Rs and cholinergic transmission (van Waarde et al., 2011). Hence, choline, as an endogenous agonist of Sig-1Rs, may be both an intracellular messenger linking GPCRs through PLD to Sig-1Rs (Figure 4F); and a paracrine signal at cholinergic synapses linking synaptic activity, through choline transporters, to Sig-1R regulation in nearby cells (Figure 4G).

We conclude that choline is an endogenous agonist of Sig-1Rs. Although we examined the consequences of activating Sig-1Rs only in the context of IP_3-evoked Ca^{2+} signals, it is likely that choline, like other agonists of Sig-1Rs, also promotes interaction of Sig-1Rs with other signaling proteins. We propose that choline may be delivered to Sig-1Rs as a paracrine reporter of activity at cholinergic synapses through choline transporters, or as an intracellular messenger from PLD activated by GPCRs (Figures 4F and 4G). The GPCRs that stimulate both PLC and PLD thereby send parallel signals to IP_3Rs: IP_3 directly activates IP_3Rs, while choline stimulates Sig-1Rs, which potentiate IP_3R activity. IP_3Rs thereby function as coincidence detectors, integrating signals from IP_3 and Sig-1Rs (Figures 3G and 4F).

STAR★METHODS

Detailed methods are provided in the online version of this paper and include the following:

- KEY RESOURCES TABLE
- CONTACT FOR REAGENT AND RESOURCE SHARING
- EXPERIMENTAL MODEL AND SUBJECT DETAILS
- METHOD DETAILS
  - Transfection of Cells
  - Radioligand Binding
  - Western Blotting
  - Microinjection and Analysis of Ca^{2+} Signals in Single Cells
  - Measurement of Ca^{2+} Signals in Cell Populations
  - Measurements of Intracellular IP_3 and Choline Concentrations
- QUANTIFICATION AND STATISTICAL ANALYSIS

SUPPLEMENTAL INFORMATION

Supplemental Information includes two figures and can be found with this article online at https://doi.org/10.1016/j.celrep.2018.12.051.

ACKNOWLEDGMENTS

This work was supported by the Wellcome Trust (101844 to C.W.T.) and NIH (R01 DA035926 to M.E.A., P30 DA 013429 to E.M.U., and R03 NS099957 to G.C.B.).

AUTHOR CONTRIBUTIONS

E.B. and C.W.T. supervised the study. E.B., S.C., G.C.B., P.Z., J.L.B., and M.A.I. performed experiments. E.B., S.C., E.M.U., M.E.A., and C.W.T. conceived and designed experiments, and analyzed and interpreted experimental data. E.B. and C.W.T. wrote the manuscript with contributions from all authors. All authors commented on the manuscript.

DECLARATION OF INTERESTS

The authors declare no competing interests.

Received: July 31, 2018
Revised: October 11, 2018
Accepted: December 11, 2018
Published: January 8, 2019

REFERENCES

Alon, A., Schmidt, H., Zheng, S., and Kruse, A.C. (2017). Structural perspectives on sigma-1 receptor function. Adv. Exp. Med. Biol. *964*, 5–13.

Aydar, E., Palmer, C.P., Klyachko, V.A., and Jackson, M.B. (2002). The sigma receptor as a ligand-regulated auxiliary potassium channel subunit. Neuron *34*, 399–410.

Brailoiu, E., Churamani, D., Cai, X., Schrlau, M.G., Brailoiu, G.C., Gao, X., Hooper, R., Boulware, M.J., Dun, N.J., Marchant, J.S., and Patel, S. (2009). Essential requirement for two-pore channel 1 in NAADP-mediated calcium signaling. J. Cell Biol. *186*, 201–209.

Brailoiu, G.C., Deliu, E., Console-Bram, L.M., Soboloff, J., Abood, M.E., Unterwald, E.M., and Brailoiu, E. (2016). Cocaine inhibits store-operated Ca^{2+} entry in brain microvascular endothelial cells: critical role for sigma-1 receptors. Biochem. J. *473*, 1–5.

Cheng, Y., and Prusoff, W.H. (1973). Relationship between the inhibition constant (K_i) and the concentration of inhibitor which causes 50 per cent inhibition (IC_{50}) of an enzymatic reaction. Biochem. Pharmacol. *22*, 3099–3108.

Corbin, K.D., and Zeisel, S.H. (2012). Choline metabolism provides novel insights into nonalcoholic fatty liver disease and its progression. Curr. Opin. Gastroenterol. *28*, 159–165.

de Costa, B.R., Bowen, W.D., Hellewell, S.B., Walker, J.M., Thurkauf, A., Jacobson, A.E., and Rice, K.C. (1989). Synthesis and evaluation of optically pure $[^3H]$-(+)-pentazocine, a highly potent and selective radioligand for sigma receptors. FEBS Lett. *251*, 53–58.

Exton, J.H. (1999). Regulation of phospholipase D. Biochim. Biophys. Acta *1439*, 121–133.

Fontanilla, D., Johannessen, M., Hajipour, A.R., Cozzi, N.V., Jackson, M.B., and Ruoho, A.E. (2009). The hallucinogen *N*,*N*-dimethyltryptamine (DMT) is an endogenous sigma-1 receptor regulator. Science *323*, 934–937.

Glennon, R.A. (2005). Pharmacophore identification for sigma-1 (sigma1) receptor binding: application of the "deconstruction-reconstruction-elaboration" approach. Mini Rev. Med. Chem. *5*, 927–940.

Glennon, M.C., Bird, G.S.J., Kwan, C.-Y., and Putney, J.W., Jr. (1992). Actions of vasopressin and the Ca^{2+}-ATPase inhibitor, thapsigargin, on Ca^{2+} signaling in hepatocytes. J. Biol. Chem. *267*, 8230–8233.

Gromek, K.A., Suchy, F.P., Meddaugh, H.R., Wrobel, R.L., LaPointe, L.M., Chu, U.B., Primm, J.G., Ruoho, A.E., Senes, A., and Fox, B.G. (2014). The oligomeric states of the purified sigma-1 receptor are stabilized by ligands. J. Biol. Chem. *289*, 20333–20344.

Grynkiewicz, G., Poenie, M., and Tsien, R.Y. (1985). A new generation of Ca^{2+} indicators with greatly improved fluorescence properties. J. Biol. Chem. *260*, 3440–3450.

Gueguinou, M., Crottès, D., Chantôme, A., Rapetti-Mauss, R., Potier-Cartereau, M., Clarysse, L., Girault, A., Fourbon, Y., Jézéquel, P., Guérin-Charbonnel, C., et al. (2017). The SigmaR1 chaperone drives breast and colorectal cancer cell migration by tuning SK3-dependent Ca^{2+} homeostasis. Oncogene *36*, 3640–3647.

Guse, A.H., Berg, I., da Silva, C.P., Potter, B.V., and Mayr, G.W. (1997). Ca^{2+} entry induced by cyclic ADP-ribose in intact T-lymphocytes. J. Biol. Chem. *272*, 8546–8550.

Haga, T. (2014). Molecular properties of the high-affinity choline transporter CHT1. J. Biochem. *156*, 181–194.

Hamprecht, B., Glaser, T., Reiser, G., Bayer, E., and Propst, F. (1985). Culture and characteristics of hormone-responsive neuroblastoma X glioma hybrid cells. Methods Enzymol. *109*, 316–341.

Hayashi, T., and Su, T.P. (2007). Sigma-1 receptor chaperones at the ER-mitochondrion interface regulate Ca^{2+} signaling and cell survival. Cell *131*, 596–610.

Hayashi, T., Maurice, T., and Su, T.P. (2000). Ca^{2+} signaling via sigma$_1$-receptors: novel regulatory mechanism affecting intracellular Ca^{2+} concentration. J. Pharmacol. Exp. Ther. *293*, 788–798.

Hong, W., Nuwayhid, S.J., and Werling, L.L. (2004). Modulation of bradykinin-induced calcium changes in SH-SY5Y cells by neurosteroids and sigma receptor ligands via a shared mechanism. Synapse *54*, 102–110.

Konieczny, V., Tovey, S.C., Mataragka, S., Prole, D.L., and Taylor, C.W. (2017). Cyclic AMP recruits a discrete intracellular Ca^{2+} store by unmasking hypersensitive IP_3 receptors. Cell Rep. *18*, 711–722.

Machová, E., O'Regan, S., Newcombe, J., Meunier, F.M., Prentice, J., Dove, R., Lisá, V., and Dolezal, V. (2009). Detection of choline transporter-like 1 protein CTL1 in neuroblastoma x glioma cells and in the CNS, and its role in choline uptake. J. Neurochem. *110*, 1297–1309.

Maurice, T., and Su, T.P. (2009). The pharmacology of sigma-1 receptors. Pharmacol. Ther. *124*, 195–206.

Mishra, A.K., Mavlyutov, T., Singh, D.R., Biener, G., Yang, J., Oliver, J.A., Ruoho, A., and Raicu, V. (2015). The sigma-1 receptors are present in monomeric and oligomeric forms in living cells in the presence and absence of ligands. Biochem. J. *466*, 263–271.

Monnet, F.P., and Maurice, T. (2006). The sigma1 protein as a target for the non-genomic effects of neuro(active)steroids: molecular, physiological, and behavioral aspects. J. Pharmacol. Sci. *100*, 93–118.

Ossa, F., Schnell, J.R., and Ortega-Roldan, J.L. (2017). A review of the human sigma-1 receptor structure. Adv. Exp. Med. Biol. *964*, 15–29.

Pelech, S.L., and Vance, D.E. (1984). Regulation of phosphatidylcholine biosynthesis. Biochim. Biophys. Acta *779*, 217–251.

Picciotto, M.R., Higley, M.J., and Mineur, Y.S. (2012). Acetylcholine as a neuromodulator: cholinergic signaling shapes nervous system function and behavior. Neuron *76*, 116–129.

Ramachandran, S., Chu, U.B., Mavlyutov, T.A., Pal, A., Pyne, S., and Ruoho, A.E. (2009). The sigma1 receptor interacts with N-alkyl amines and endogenous sphingolipids. Eur. J. Pharmacol. *609*, 19–26.

Rouzaire-Dubois, B., and Dubois, J.M. (1997). A proton pump contributes to neuroblastoma x glioma cell membrane potentials. Pflugers Arch. *434*, 750–755.

Sak, K., Samuel, K., Kelve, M., and Webb, T.E. (2001). Pharmacological characterisation of pyrimidinoceptor responses in NG108-15 cells. Eur. J. Pharmacol. *415*, 127–133.

Sarter, M., and Parikh, V. (2005). Choline transporters, cholinergic transmission and cognition. Nat. Rev. Neurosci. *6*, 48–56.

Schmidt, H.R., Zheng, S., Gurpinar, E., Koehl, A., Manglik, A., and Kruse, A.C. (2016). Crystal structure of the human $\sigma1$ receptor. Nature *532*, 527–530.

Selvy, P.E., Lavieri, R.R., Lindsley, C.W., and Brown, H.A. (2011). Phospholipase D: enzymology, functionality, and chemical modulation. Chem. Rev. *111*, 6064–6119.

Shioda, N., Ishikawa, K., Tagashira, H., Ishizuka, T., Yawo, H., and Fukunaga, K. (2012). Expression of a truncated form of the endoplasmic reticulum chaperone protein, σ1 receptor, promotes mitochondrial energy depletion and apoptosis. J. Biol. Chem. *287*, 23318–23331.

Smith, S.B., and Su, T.-P. (2017). Sigma receptors: Their role in disease and as therapeutic targets. Adv. Exp. Med. Biol. *961*, 1–312.

Srivats, S., Balasuriya, D., Pasche, M., Vistal, G., Edwardson, J.M., Taylor, C.W., and Murrell-Lagnado, R.D. (2016). Sigma1 receptors inhibit store-operated Ca^{2+} entry by attenuating coupling of STIM1 to Orai1. J. Cell Biol. *213*, 65–79.

Su, T.P., Su, T.C., Nakamura, Y., and Tsai, S.Y. (2016). The sigma-1 receptor as a pluripotent modulator in living systems. Trends Pharmacol. Sci. *37*, 262–278.

Tovey, S.C., Sun, Y., and Taylor, C.W. (2006). Rapid functional assays of intracellular Ca^{2+} channels. Nat. Protoc. *1*, 259–263.

Traiffort, E., O'Regan, S., and Ruat, M. (2013). The choline transporter-like family SLC44: properties and roles in human diseases. Mol. Aspects Med. *34*, 646–654.

van Waarde, A., Ramakrishnan, N.K., Rybczynska, A.A., Elsinga, P.H., Ishiwata, K., Nijholt, I.M., Luiten, P.G., and Dierckx, R.A. (2011). The cholinergic system, sigma-1 receptors and cognition. Behav. Brain Res. *221*, 543–554.

Walker, J.M., Bowen, W.D., Walker, F.O., Matsumoto, R.R., De Costa, B., and Rice, K.C. (1990). Sigma receptors: biology and function. Pharmacol. Rev. *42*, 355–402.

Watanabe, S., Ilieva, H., Tamada, H., Nomura, H., Komine, O., Endo, F., Jin, S., Mancias, P., Kiyama, H., and Yamanaka, K. (2016). Mitochondria-associated membrane collapse is a common pathomechanism in *SIGMAR1*- and *SOD1*-linked ALS. EMBO Mol. Med. *8*, 1421–1437.

Wu, Z., and Bowen, W.D. (2008). Role of sigma-1 receptor C-terminal segment in inositol 1,4,5-trisphosphate receptor activation: constitutive enhancement of calcium signaling in MCF-7 tumor cells. J. Biol. Chem. *283*, 28198–28215.

Yamada, T., Inazu, M., Tajima, H., and Matsumiya, T. (2011). Functional expression of choline transporter-like protein 1 (CTL1) in human neuroblastoma cells and its link to acetylcholine synthesis. Neurochem. Int. *58*, 354–365.

Yano, H., Bonifazi, A., Xu, M., Guthrie, D.A., Schneck, S.N., Abramyan, A.M., Fant, A.D., Hong, W.C., Newman, A.H., and Shi, L. (2018). Pharmacological profiling of sigma 1 receptor ligands by novel receptor homomer assays. Neuropharmacology *133*, 264–275.

STAR★METHODS

KEY RESOURCES TABLE

REAGENT OR RESOURCE	SOURCE	IDENTIFIER
Antibodies		
Donkey anti-rabbit IgG-HRP (1:5000)	Santa Cruz Biotechnology Inc, Dallas, TX	Cat# sc-2313
Goat anti-mouse IgG-HRP (1:2000)	Santa Cruz Biotechnology	Cat# sc-2005
IRDye 800CW-conjugated goat anti-rabbit IgG (1:10,000)	LI-COR, Lincoln, NE	Cat# 926-32211
IRDye 680-conjugated goat anti-mouse IgG (1:10,000)	LI-COR	Cat# 926-32220
Rabbit anti-Sig-1R (1:200)	OriGene, Rockville, MD	Cat# TA302033
Rabbit anti-Sig-1R (2 μg/mL)	AbCam, Cambridge, UK	Cat# 53852
Mouse anti-GFP (1:2000)	OriGene	Cat# TA150041
Mouse anti-PLD1 (1:200)	Santa Cruz Biotechnology	Cat# sc-25512
Mouse anti-PLD2 (3 μg/mL)	Abnova Corporation, Taipei, Taiwan	Cat# H00005338
Rabbit anti-β-actin (1:2000)	Santa Cruz Biotechnology	Cat# sc-1616
Mouse anti-β-actin (1:1000)	Cell Signaling Technology, Boston, MA	Cat# 8H10D10
Mouse anti-β-actin (1:10,000)	Sigma-Aldrich, St. Louis, MO	Cat# A5441
Rabbit anti-CTL1 (1:500)	ThermoFisher, Basingstoke, UK	Cat# AB_2556158
Chemicals, Peptides, and Recombinant Proteins		
Acetylcholine chloride	Chem-IMPEX International, Wood Dale, IL	Cat# 00770
Acetylcholine chloride	Sigma-Aldrich	Cat# A6625
ATP	Sigma-Aldrich	Cat# A9187
BAPTA	Molekula, Dorset, UK	Cat# 20358510
Betaine hydrochloride	Sigma-Aldrich	Cat# 61962
BD1047 dihydrobromide	Tocris, Abingdon, UK	Cat# 0956
Bradykinin acetate salt	Sigma-Aldrich	Cat# B3259
Bovine serum albumin (BSA)	Europa Bioproducts Ltd, Cambridge, UK	Cat# EQBAH64
Choline chloride	Sigma-Aldrich	Cat# C7017
cOmplete™ protease inhibitor cocktail	Sigma-Aldrich	Cat# 4693116001
Dimethyl sulfoxide (DMSO)	Sigma-Aldrich	Cat# D2650
DMEM/F-12, GlutaMAX medium	ThermoFisher	Cat# 31331028
ECL Prime	GE Healthcare, Little Chalfont, UK	Cat# RPN2232
Fluo-8 AM	AAT Bioquest, Cambridge, UK	Cat# 21802
Fetal bovine serum (FBS)	Sigma-Aldrich	Cat# F7524, batch 094M3341
Fura-2 AM	AAT Bioquest	Cat# 21020
	ThermoFisher	Cat# F1221
G-418	ThermoFisher	Cat# 10131027
Glucose	ThermoFisher	Cat# 10141520
Haloperidol	Sigma-Aldrich	Cat# H1512
Hank's balanced salt solution (HBSS)	ThermoFisher	Cat# 21-023-CV
HEPES	Merck Millipore	Cat# 391338
Ionomycin	Apollo Scientific, Stockport, UK	Cat# 56092-81-0
Lipofectamine	ThermoFisher	Cat# 18324012
Lipofectamine RNAiMax	ThermoFisher	Cat# 13778150
Odyssey blocking buffer	LI-COR	Cat# 927-50000
Opti-MEM I	ThermoFisher	Cat# 11058-021
[^{3}H]-(+)-Pentazocine (26.9 Ci/mmol)	Perkin-Elmer, Richmond, CA	Cat# NET10560250UC
PRE-084 hydrochloride	Tocris	Cat# 0589

(*Continued on next page*)

Continued

REAGENT OR RESOURCE	SOURCE	IDENTIFIER
Phosphocholine chloride	Tokyo Chemical Industry, Japan	Cat# P0834
Pluronic F127	Sigma-Aldrich	Cat# P2443
Polyethyleneimine	Sigma-Aldrich	Cat# P3143
RPMI medium	ThermoFisher	Cat# MT10041CM
(+)SKF-10047 hydrochloride	Tocris	Cat# 1079
Sodium fluoride	Sigma-Aldrich	Cat# S7920
Sodium orthovanadate	Sigma-Aldrich	Cat# S6508
TurboFectin 8.0	OriGene	Cat# TF81001
Tris base	ThermoFisher	Cat# BP152-1
Triton X-100	Sigma-Aldrich	Cat# T8787
Tween-20	Sigma-Aldrich	Cat# T5927
Critical Commercial Assays		
BCA protein assay kit (Pierce)	ThermoFisher	Cat# 23225
Choline assay kit	BioVision, Mountain View, CA	Cat# K615-100
IP_3 assay kit	DiscoveRx, Fremont, CA	Cat# 90-0037
Experimental Models: Cell Lines		
NG108-15 cells	American Type Culture Collection (ATCC), Manassas, VA	Cat# ATCC HB-12317
MCF7 cells	ATCC	Cat# ATCC HTB-22
Neuro-2A cells	ATCC	Cat# ATCC CCL-131
Recombinant DNA		
Human Sig-1R-GFP in pCMV6-AC-GFP	OriGene	Cat# RG201206
RFP-tagged shRNA (HuSH, 29-mer shRNA in pRFP-C-RS) against human Sig-1R [GAGTAT GTGCTGCTCTTCGGCACCGCCTT]	OriGene	Cat# TF311012<br>FI344041
RFP-tagged shRNA (HuSH, 29-mer shRNA in pRFP-C-RS) against rat PLD1 [GCCTCTATCG CCAACTTCACCGCCGTAAT]	OriGene	Cat# TF711124<br>FI744500
RFP-tagged shRNA (HuSH, 29-mer shRNA in pRFP-C-RS) against rat PLD2 [GGAGACTGG ACATTATGCTCAAGAGGAAG]	OriGene	Cat# TF711696<br>FI746786
RFP-tagged scrambled shRNA (HuSH, 29-mer scrambled shRNA in pRFP-C-RS)	OriGene	Cat# TF311012<br>TR30015
Silencer siRNA (3 different 21-bp siRNA) against rat CTL1 (SLC44A1)	ThermoFisher	Cat# 192756<br>Cat# 192757<br>Cat# 55087
Control Silencer siRNA	ThermoFisher	Cat# AM4611
Software and Algorithms		
Prism 5, version 5	GraphPad, La Jolla	https://www.graphpad.com/
GeneTools, version 4	Syngene, Cambridge, UK	https://www.syngene.com/
Odyssey, version 3	LI-COR	https://www.licor.com/
SoftMax Pro, version 7	Molecular Devices, San Jose, CA	https://www.moleculardevices.com/
NIS-Elements AR 3.1	Nikon, Melville, NY	https://www.nikon.com/

CONTACT FOR REAGENT AND RESOURCE SHARING

Further information and requests for resources and reagents should be directed to and will be fulfilled by the Lead Contact, Colin W. Taylor (cwt1000@cam.ac.uk).

EXPERIMENTAL MODEL AND SUBJECT DETAILS

The NG108-15 cell line (ATCC) is a somatic hybrid derived from a mouse neuroblastoma and rat glioma. NG108-15 cells were grown in DMEM/F12 with 10% fetal bovine serum (FBS). MCF7 cells (ATCC) were derived from a human metastatic mammary tumor. These cells were grown in RPMI with 10% FBS. Neuro-2A cells (ATCC), which were used only for heterologous expression of Sig-1R-GFP for radioligand binding analyses, were derived from a mouse neuroblastoma. Neuro-2A cells were grown in DMEM containing 10% FBS, and further supplemented with G-418 (100 μg/mL) for the cells stably expressing Sig-1R-GFP. We have not established the sex of the animals from which NG108-15 and Neuro-2A cells were derived. All cells were grown in humidified air at 37°C with 5% CO_2. Cells were passaged when they reached around 80% confluence. The authenticity of the cell lines was not confirmed, but screening established that all cells were free of mycoplasma.

METHOD DETAILS

Transfection of Cells

Cells were transiently transfected using either TurboFectin 8.0 or electroporation. For the former, plasmid DNA was added to TurboFectin 8.0 in OptiMEM I (TurboFectin:DNA, 3:1), incubated (15-30 min, 20°C), and the complex was then added to cells in 6-well plates (1-1.5 μg DNA/well) in complete medium, and incubated for 24-48 h. For electroporation, cells (80%–90% confluent in a T75 flask) were scraped into culture medium, centrifuged (150 x*g*, 5 min), and resuspended in Opti-MEM I (2×10^6 cells/mL). Cells (500 μL) were transferred to electroporation cuvettes (800 μL, 4-mm gap; Eppendorf, Hamburg, Germany) with plasmid DNA (5-10 μg/cuvette) and the cells were subjected to electroporation using a GenePulser Xcell (BioRad, 200-250V, 700-900 μF, 18-20 ms). Transfected cells were plated in Opti-MEM I in 6-well plates, FBS (10%) was added after 4 h, and the medium was replaced after 24 h.

Neuro-2A cells stably expressing Sig-1R-GFP were generated by transfecting cells with plasmid encoding human Sig-1R-GFP using Lipofectamine. Cells were grown in medium containing G418 (400 μg/mL), and after 2 weeks resistant colonies were selected and propagated. Stable cell lines with intermediate levels of Sig-1R-GFP expression (determined by fluorescence microscopy) were identified and then maintained in DMEM supplemented with FCS (10%) and G418 (100 μg/mL).

For expression of human Sig-1R-GFP, cells grown in 6-well plates were transfected with 1-1.5 μg DNA/well. To reduce expression of Sig-1R or PLDs, RFP-tagged shRNA constructs were used. Each set of constructs included four different 29-mer targeting shRNA in a pRFP-C-RS plasmid. Using methods reported previously (Brailoiu et al., 2016), we used western blotting to assess the ability of each individual construct to reduce expression of its target protein (Sig-1R, PLD1 or PLD2). The most effective shRNA construct from each set was used for the experiments described here. The constructs were used individually for Sig-1R knockdown (2 μg/mL) or as a pair for knockdown of PLD1 and PLD2 (1 μg/mL of each). The same scrambled RFP-shRNA construct (2 μg/mL) was used as a control for all shRNA analyses.

Lipofectamine RNAiMax was used to transfect cells simultaneously with three different siRNAs against CTL1 (50 nM of each) to reduce CTL1 expression. A siRNA with no known target in mammalian genomes (150 nM) was used as a control for the siRNA experiments (Silencer control, ThermoFisher). Cells were used 24-48 h after transfection.

Radioligand Binding

Membranes were prepared from Neuro-2A cells stably expressing Sig-1R-GFP (Wu and Bowen, 2008). Cells ($\sim 1.7 \times 10^8$) were harvested (500 x*g*, 5 min) in phosphate-buffered saline (PBS) containing EGTA (1 mM), homogenized in cold medium (10 mL; 50 mM Tris-HCl, 320 mM sucrose, 2 mM EDTA, 5 mM $MgCl_2$, pH 7.4), centrifuged (50,000 x*g*, 4°C, 10 min), the pellet was then resuspended by homogenization (2 mg protein/mL) in binding medium (50 mM Tris-HCl, 1 mM EDTA, 3 mM $MgCl_2$, pH 7.4) and stored at −80°C. Binding assays (final volume 500 μL) were performed in glass tubes with binding medium containing BSA (5 mg/mL), [^{3}H](+)-pentazocine (5 nM, 26.9 Ci/mmol), competing ligands and membranes (100 μg). After 1 h at 30°C, bound ligand was recovered by rapid filtration through Whatman GF/C filters pre-soaked in polyethyleneimine (0.1%, 2 h), the filters were washed twice, and their radioactivity was determined by liquid scintillation counting. Non-specific binding was determined in the presence of 5 μM haloperidol.

Western Blotting

Lysates were prepared from cells 48 h after transfection. Cells were collected (150 x*g*, 5 min) and lysed (1 h, 4°C) in medium comprising: NaCl (50 mM), Tris (20 mM), Mg acetate (10 mM), Triton X-100 (1%, v/v), cOmplete protease inhibitor mixture, Na orthovanadate (1 mM) and Na fluoride (5 mM), pH 7.3. After centrifugation (14,000 x*g*, 15 min), the supernatant was collected and its protein concentration determined using a BCA assay kit. Cell lysates, which were used immediately or after storage at −80°C, were subject to SDS-PAGE using Mini-PROTEAN TGX 4%–20% gels (BioRad, Hercules, CA) or NuPAGE 4%–12% Bis-Tris gels (Invitrogen, Paisley, UK). Proteins were transferred to Odyssey nitrocellulose membranes (LI-COR Biosciences) or PVDF membranes (iBlot, Invitrogen**)**. Membranes were washed and blocked (1 h, 20°C) with Odyssey blocking buffer or TBST (137 mM NaCl, 20 mM Tris, 0.1% Tween-20, pH 7.6) supplemented with 5% (w/v) BSA. Membranes were incubated (12 h, 4°C) with primary antibodies in TBST and 1% BSA, washed with TBST (3 × 5 min), incubated with secondary antibodies in TBST and 1% BSA (1 h, 20°C), and then washed with TBST. Bands were visualized by infrared emission (LI-COR Infrared Imager, resolution 169 μm, intensity 4.5-6)

or by incubation with HRP-conjugated secondary antibodies (1 h), followed by washing and detection with ECL Prime. Densitometric analysis used Odyssey or GeneTools software, or ImageJ (NIH, Bethesda, USA). The antibodies used and their dilutions are listed in the Key Resources Table.

Microinjection and Analysis of Ca^{2+} Signals in Single Cells

For measurements of $[Ca^{2+}]_i$ in single Fura-2-loaded cells grown on glass coverslips (#1.5, 25-mm diameter, Warner Instruments), cells were incubated with Fura-2 AM (5 μM, 45 min, 20°C) in Hanks' balanced salt solution (HBSS), washed 3 times, and incubated for a further 45 min before experiments (Brailoiu et al., 2009). Fluorescence images (alternate excitation at 340 and 380 nm; emission at 510 nm) were acquired at 0.25 Hz using an inverted Nikon Eclipse Ti microscope with a Perfect Focus System and a CoolSnap HQ2 CCD camera (Photometrics Scientific). Images were acquired and analyzed using NIS-Elements AR 3.1 software (Nikon). After correction for background, determined from an area outside the cell, fluorescence ratios (F_{340}/F_{380}) were calibrated to $[Ca^{2+}]_i$ (Grynkiewicz et al., 1985). Injections were performed using Femtotips II, InjectMan N I2 and FemtoJet systems (Eppendorf) (Brailoiu et al., 2009). Pipettes were back-filled with intracellular solution (110 mM KCl, 10 mM NaCl, 20 mM HEPES, pH 7.2) (Guse et al., 1997) and appropriate drugs. The injection time was 0.4 s at 60 hPa with a compensation pressure of 20 hPa in order to inject ~1% of the cell volume.

Measurement of Ca^{2+} Signals in Cell Populations

For measurements of $[Ca^{2+}]_i$ in cell populations, confluent cultures of cells in 96-well plates were loaded with Fluo-8 by incubation with Fluo-8 AM (2 μM, 30 min, 20°C) in HEPES-buffered saline (HBS) supplemented with 0.02% pluronic acid. The medium was then replaced with HBS, and after 30 min at 20°C to allow de-esterification of the indicator, fluorescence was recorded using a FlexStation III plate-reader (MDS Analytical Devices, Wokingham, UK) (Konieczny et al., 2017; Tovey et al., 2006). Fluorescence was captured and processed using SoftMax Pro software. All measurements were performed in HBS at 20°C. HBS comprised: 135 mM NaCl, 5.9 mM KCl, 1.2 mM $MgCl_2$, 1.5 mM $CaCl_2$, 11.5 mM glucose, 11.6 mM HEPES, pH 7.3. Fluorescence was recorded at 1.44 s intervals, with excitation at 485 nm and emission at 525 nm. The minimal (F_{min}, Ca^{2+}-free indicator) and maximal (F_{max}, Ca^{2+}-saturated indicator) fluorescence values were determined from several parallel wells in each plate after addition of Triton X-100 (0.1%) with either BAPTA (10 mM, for F_{min}) or $CaCl_2$ (10 mM, for F_{max}). Fluorescence values (F) were then calibrated to $[Ca^{2+}]_i$ from:

$$[Ca^{2+}]_i = K_D \times \frac{F - F_{min}}{F_{max} - F}$$

The K_D of fluo-8 was assumed to be 389 nM.

Measurements of Intracellular IP_3 and Choline Concentrations

NG108-15 cells (10^{10} cells) in HBSS (0.5 mL, 20°C) were stimulated with ATP and the reaction was terminated by addition of cold $HClO_4$ (1 mL, 0.75 M). After centrifugation (2000 × *g*, 5 min, 4°C), the supernatant was removed, PBS (270 μL) was added, and the mixture was sonicated. After centrifugation (15,000 × *g*, 10 min), assay kits were used to determine the amounts of choline (BioVision Inc.) and IP_3 (DiscoveRx) in the supernatant, according to the manufacturer's instructions. A volume of 2.5 pL for an NG108-15 cell (Rouzaire-Dubois and Dubois, 1997) was used to calculate intracellular concentrations of IP_3 and choline.

QUANTIFICATION AND STATISTICAL ANALYSIS

For analyses of radioligand binding results, each equilibrium competition-binding curve was fitted to a logistic equation (GraphPad Prism, version 5), from which the half-maximal inhibitory concentration (IC_{50}) and Hill coefficient (*h*) were determined. The IC_{50} value, $[^3H]$(+)-pentazocine concentration (5 nM) and K_d of (+)pentazocine for Sig-1R (5.5 nM) (de Costa et al., 1989) were used to calculate K_i values (K_i is the K_d determined by equilibrium competition binding) (Cheng and Prusoff, 1973). The negative logarithms of these individual K_i values (pK_i) were pooled for statistical analysis. All results are presented as means ± SD or SEM, as appropriate, from *n* independent analyses. ANOVA, followed by Dunnett's, Bonferroni or Tukey tests, was used to evaluate differences between groups (GraphPad Prism, version 5). $p < 0.05$ was considered significant. The tests used are reported in the figure legends.

Cell Reports, Volume *26*

Supplemental Information

Choline Is an Intracellular Messenger Linking Extracellular Stimuli to IP_3-Evoked Ca^{2+} Signals through Sigma-1 Receptors

Eugen Brailoiu, Sumita Chakraborty, G. Cristina Brailoiu, Pingwei Zhao, Jeffrey L. Barr, Marc A. Ilies, Ellen M. Unterwald, Mary E. Abood, and Colin W. Taylor

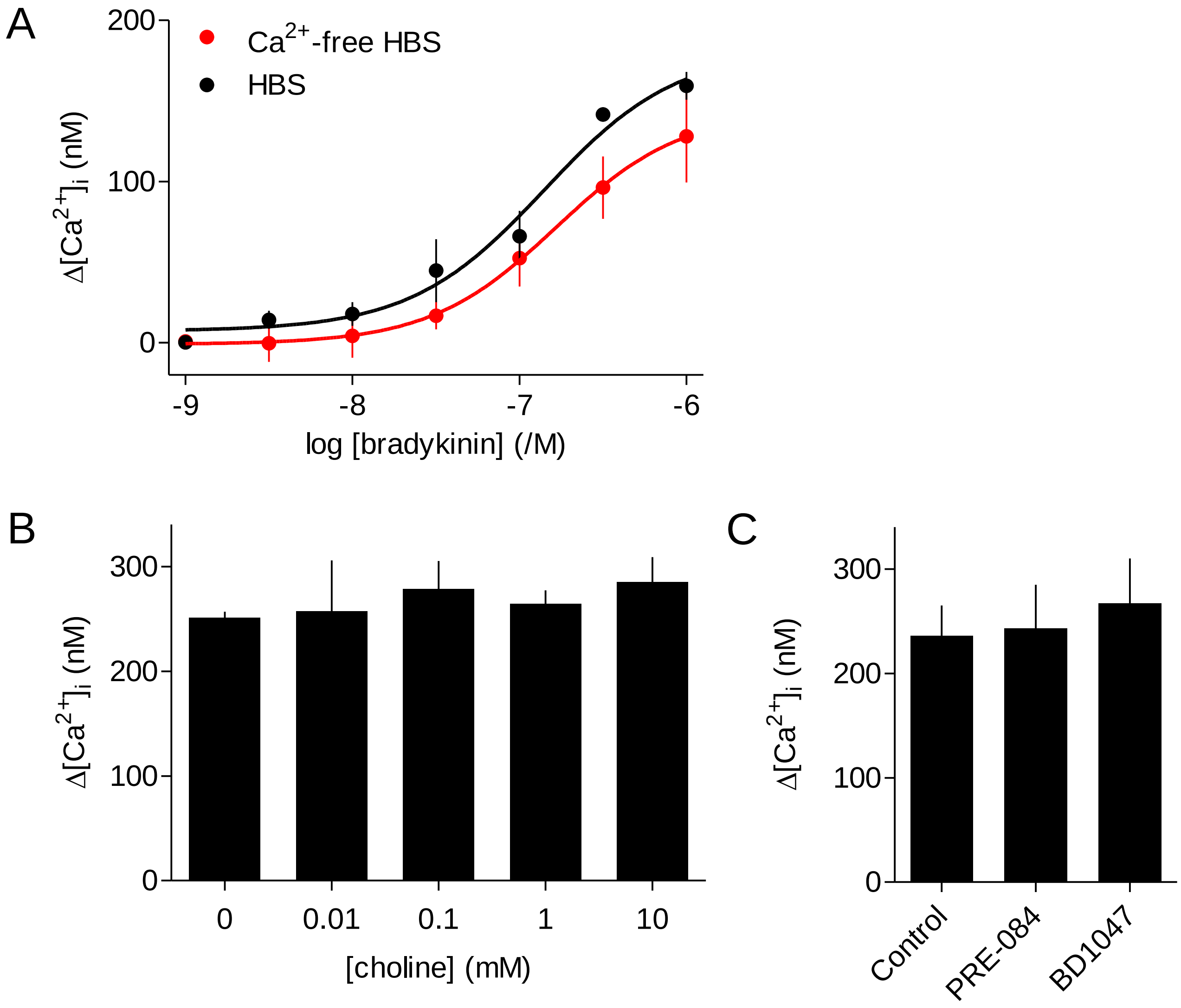

Figure S1. Bradykinin Stimulates Ca^{2+} Release From the Intracellular Stores of NG108-15 Cells.
Related to Figures 1 and 2

(A) NG108-15 cells were stimulated with the indicated concentrations of bradykinin in HBS or Ca^{2+}-free HBS. Results (means ± SEM, n = 3, with duplicate determination) show $\Delta[Ca^{2+}]_i$.
(B,C) Intact cells were treated with the indicated concentrations of choline (1 h, 20°C) (B) or incubated (3 h, 37°C) with PRE-084 (25 μM) or BD1047 (25 μM) (C) before addition of ionomycin (5 μM) in Ca^{2+}-free HBS to assess the Ca^{2+} content of the intracellular stores. Results (mean ± SEM, n = 3) show $\Delta[Ca^{2+}]_i$ evoked by ionomycin.

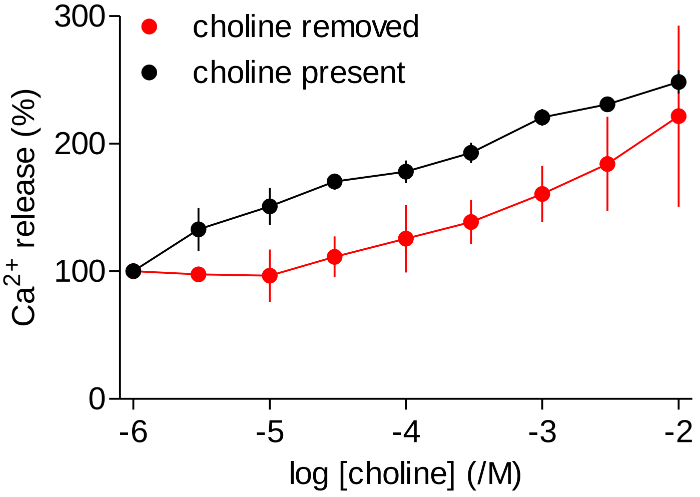

Figure S2. Intracellular Choline Potentiates Bradykinin-Evoked Ca^{2+} Signals. Related to Figure 4.

NG108-15 cells were incubated (1 h) with the indicated concentrations of choline in HBS before recording the peak Ca^{2+} signals evoked by bradykinin (1 µM), which was added in either the continued presence of choline or immediately after its removal. Responses (mean ± SEM, *n* = 3 plates with duplicate determinations; several error bars are smaller than the symbols) are expressed as a percentage of the matched response to bradykinin without choline (100%).
